# Impact of silver nanoparticles on the nutritional properties of *Arthrospira platensis*

**DOI:** 10.7717/peerj.13972

**Published:** 2022-10-10

**Authors:** Sharolynne Xiao Tong Liang, Sinouvassane Djearamane, Anto Cordelia Tanislaus Antony Dhanapal, Ling Shing Wong

**Affiliations:** 1 Department of Biomedical Science, Faculty of Science, Universiti Tunku Abdul Rahman, Kampar, Perak, Malaysia; 2 Department of Chemical Science, Faculty of Science, Universiti Tunku Abdul Rahman, Kampar, Perak, Malaysia; 3 Faculty of Health and Life Sciences, INTI International University, Nilai, Negeri Sembilan, Malaysia

**Keywords:** Silver nanoparticles, Cellular interaction, Nutrients, *Arthrospira platensis*

## Abstract

**Background:**

*Arthrospira platensis* is farmed worldwide due to its nutrient-rich properties and provides multiple benefits to human health. However, the wide usage of silver nanoparticles (Ag NPs) causes pollution which may affect the nutritional quality of *A. platensis*. Hence, this study aimed to investigate the interaction and accumulation of Ag NPs on *A. platensis*, and determine the changes in biomass and nutritional value of *A. platensis* due to the exposure to Ag NPs.

**Methods:**

The interaction and accumulation of Ag NPs on *A. platensis* were examined through Fourier transformed infrared (FTIR) spectroscopy and scanning electron microscope (SEM). The loss in biomass together with the macromolecules, pigments, and phenolic compounds of *A. platensis* was investigated upon treating with various concentrations of Ag NPs (5, 10, 25, 50 and 100 µg/mL) for 24, 48, 72 and 96 h.

**Results:**

The results showed that the treatment of *A. platensis* with Ag NPs caused a dose and time-dependent reduction in biomass, macronutrients, pigments and phenolic compounds. The highest detrimental effects were found at 96 h with the reported values of 65.71 ± 2.79%, 67.21 ± 3.98%, 48.99 ± 4.39% and 59.62 ± 3.96% reduction in biomass, proteins, carbohydrates and lipids, respectively, along with 82.99 ± 7.81%, 67.55 ± 2.63%, 75.03 ± 1.55%, and 63.43 ± 2.89% loss in chlorophyll-*a*, carotenoids, C-phycocyanin, and total phenolic compounds of *A. platensis* for 100 µg/mL of Ag NPs. The EDX analysis confirmed the surface accumulation of Ag NPs on *Arthrospira* cells, while SEM images evidenced the surface alterations and damage of the treated cells. The functional groups such as hydroxyl, amine, methyl, amide I, amide II, carboxyl, carbonyl and phosphate groups from the cell wall of the *A. platensis* were identified to be possibly involved in the interaction of Ag NPs with *A. platensis*.

**Conclusion:**

The study confirmed that the exposure of Ag NPs is detrimental to *A. platensis* where the interaction and accumulation of Ag NPs on *A. platensis* caused reduction in biomass, macromolecules, pigments, and total phenolic compounds.

## Introduction

Nanotechnology has been used in various fields such as in electronics, biological sensors, and water treatments (Sirelkhatim et al., 2015; Syafiuddin et al., 2018; Dhanasegaran et al., 2022). The applications of engineered nanomaterials (ENMs) result in the release of huge amount of nanoparticles (NPs) into the environment. The effluents from wastewater treatment plants and incineration of waste products are reported to be the major entry points of NPs into the aquatic environment (Dalai et al., 2012; Li & Lenhart, 2012; Liang et al., 2020). Silver nanoparticles (Ag NPs) are estimated to have a worldwide production of about 360 to 450 tons per year by 2025 and will increase to produce around 800 tons per year in the future (Prabhu & Dhulappa, 2022). According to the Allied Market Research report released in 2022, Ag NPs global market was valued $1.5 billion in the year of 2020, and is expected to reach $6.6 billion by 2030, with a compound annual growth rate of 15.6% from year 2021 to 2030 (Moon, Narunem & Prasad, 2022). This implies that Ag NPs are widely utilized on a global scale.

Photosynthetic microbes such as microalgae and cyanobacteria play an important role in the ecosystem, providing biomass as the basic food source for the food chain in the aquatic system. Cyanobacterium *Arthrospira platensis* is known as an important photosynthetic microbe which serves as food and food supplement due to its high nutritional value. *A. platensis* has been commercially produced for over 30 years and used in fish feed, vitamin supplements, food dyes, aquaculture, pharmaceuticals, and nutraceuticals products (Abdulqader, Barsanti & Tredici, 2000; de Marco Castro, Shannon & Abu-Ghannam, 2019).

Photosynthetic microbes are sensitive to environmental toxicants. While they are the most vulnerable aquatic organisms to NPs, the toxicity effects might bring implications on higher organisms through the food chain (Neale et al., 2015; Tang et al., 2018; Zhang et al., 2016). The nutritional quality of photosynthetic microbes in the water bodies will be affected by the contamination of ENMs (Lohse et al., 2017; Lone et al., 2013). The present study investigated the interaction and accumulation of Ag NPs in *A. platensis*, and determined the changes in biomass production due to Ag NPs and the effect of Ag NPs to macromolecules, pigments, and phenolic compounds in *A. platensis*.

## Methodology

### Cultivation of *A. platensis*

The cyanobacterium *A. platensis* culture was obtained from University of Texas Culture Collection, Austin, Texas, United States of America (UTEX number: LB1926). The cyanobacterial cells were cultured using *Spirulina* medium and maintained in conical flasks under 1,200 lux illumination using cool white fluorescent lamp with 16 h light and 8 h dark conditions, at room temperature (21–23 °C).

### Characterization of Ag NPs

Ag NPs powder (particle size <100 nm) was purchased from Sigma-Aldrich, Malaysia. Scanning electron microscope with energy dispersive X-ray (SEM-EDX, JSM-6701F, Joel, Japan), and X-ray diffractometer (XRD, 600, Shimadzu, Japan) were used to characterize the nanoparticles. The particle size was determined using SEM operated at acceleration voltage of 4.0 kV with working distance of 6.0 mm. The chemical composition was confirmed by EDX with an acceleration voltage of 10 kV. The crystalline nature of Ag NPs was analyzed using XRD operated at a voltage of 40 kV and current of 30 mA with Cu radiation *λ* = 1.5406 in the scan range of 2θ = 20°–80°.

### Exposure of *A. platensis* to Ag NPs

Ag NPs stock solution (200 μg/mL) was prepared by adding 100 mg of Ag NPs powder to 500 mL of *Spirulina* medium. The mixture was sonicated at 45 kHz for 1 h to obtain the homogenous suspension of Ag NPs. Then the five different working concentrations of Ag NPs (5, 10, 25, 50, and 100 μg/mL) were prepared by diluting the stock solution in *Spirulina* culture medium. The *A. platensis* cell suspension at the exponential phase (Day 7) with an initial density of 0.6 at OD_560_ was exposed to all five working concentrations of Ag NPs for different time intervals (24, 48, 72, and 96 h). The cell suspension without NPs treatment was served as the negative control.

### Determination of cellular interaction of Ag NPs on *A. platensis*

Attenuated total reflectance Fourier transformed infrared (ATR-FTIR, Spectrum Two, Perkin-Elmer, United States of America) spectroscopy was carried out to confirm the involvement of functional groups from the cell wall of *A. platensis* which were believed to be interacted with Ag NPs. A volume of 25 mL cyanobacterial suspension treated with NPs was centrifuged for 10 min at 5,000 rpm and the isolated pellet was washed twice with 1X phosphate buffered saline (PBS) and distilled water. The cells were freeze dried to remove moisture and subjected to ATR-FTIR analysis over the range of 4,000 to 400 cm^−1^ using the reflection technique.

SEM-EDX was carried out to identify the cellular accumulation of Ag NPs in the biomass of *A. platensis* and to study the morphological damages on cyanobacterial cells resulted from the treatment of Ag NPs. A volume of 25 mL Ag NPs treated cyanobacterial cell suspension was centrifuged at 5,000 rpm for 10 min and the pelleted cells were washed with 0.1X PBS and distilled water for twice and freeze dried. The freeze-dried cyanobacterial cells were subjected to SEM-EDX analysis.

### Effects of Ag NPs to biomass of *A. platensis*

The changes in the biomass of *A. platensis* due to the presence of Ag NPs (5, 10, 25, 50, and 100 µg/mL) over 24, 48, 72, and 96 h were determined by measuring the optical density of the culture at 560 nm (OD_560_) using a spectrophotometer (Genesys 10S UV-Vis, Thermo Scientific, Waltham, MA, USA). The cell culture without the presence of Ag NPs was used as negative control. The growth medium without the cells was used as blank for the tests. The optical density of medium with Ag NPs at OD_560_ was measured and subtracted from the respective test readings to minimize the interference from Ag NPs (Eq. (1)). The percentage of change in biomass due to the presence of Ag NPs was calculated using Eq. (2).

(1) $$\rm{OD \; of \; the \; culture \;at \; 560 \; nm={OD_1}-{OD_0}}$$

where

OD_0_ = OD of the medium with Ag NPs only

OD_1_ = OD of cell culture with Ag NPs

(2) $$\eqalign{
  & \% \;{\rm{change}}\;{\rm{in}}\;{\rm{biomass}} = ({\rm{O}}{{\rm{D}}_{{\rm{560}}}}\;{\rm{of}}\;{\rm{negative}}\;{\rm{control - O}}{{\rm{D}}_{{\rm{560}}}}\;{\rm{of}}\;{\rm{cell}}\;{\rm{culture}}) \times   \cr 
  & \quad 100/({\rm{O}}{{\rm{D}}_{{\rm{560}}}}\;{\rm{of}}\;{\rm{negative}}\;{\rm{control}}) \cr} $$

### Effects of Ag NPs to proteins

The proteins from the cells were extracted using alkali method. A volume of 10 mL of treated and untreated cyanobacterial suspensions were centrifuged at 5,000 rpm for 10 min and supernatant was discarded. A volume of 4.5 mL of 0.5 N sodium hydroxide (NaOH) was added to the pellet followed by an incubation in water bath at 80 °C for 10 min. The mixture was centrifuged again after incubation and the protein content in the supernatant was estimated using Lowry method (Anusha, Chingangbam & Sibi, 2017). A volume of 1 mL of supernatant was transferred into a new tube and 2 mL of Lowry solution (Lowry reagent A:B:C = 48:1:1) was added and incubated for 10 min at room temperature. After incubation, 0.2 mL of 1 N Folin-Ciocalteau phenol reagent (FCR) was added into mixture, vortexed and incubated in dark for 30 min. The absorbance of the mixture was measured using microplate reader (FLUOstar Omega, BMG labtech, Germany) at 600 nm (Tan et al., 2020). Bovine serum albumin (BSA) was used to generate a standard curve to estimate the protein content in the supernatant. The percentage change of proteins due to the presence of Ag NPs was calculated using Eq. (3).

(3) $$\rm{{\%}\;change\;in\;protein\;content = ({P_0} - {P_1}) \times 100\%/{P_0}}$$

where

P_0_ = Proteins in untreated cells

P_1_ = Proteins in treated cells

### Effects of Ag NPs to carbohydrates

The extraction of carbohydrates from the cells was performed using HCl extraction. A volume of 10 mL of cyanobacterial sample was centrifuged at 5,000 rpm for 10 min and the supernatant was discarded. A volume of 5 mL of 2.5 N HCl was added to the pellet and incubated in 90 °C water bath for 3 h and cooled to room temperature (Sharma et al., 2019). Sodium carbonate (Na_2_CO_3_) was added to neutralize the sample until no effervescence was observed. The sample was then topped up to 50 mL with distilled water and centrifuged at 5,000 rpm for 10 min to settle down the solid particles (Agrawal, Minj & Rani, 2015). The carbohydrate content in the supernatant was estimated using the phenol-sulfuric acid method. A volume of 1 mL of supernatant was transferred into a new tube, 1 mL of distilled water and 50 μL of 80% phenol was added and the mixture was vortexed. A volume of 5 mL of concentrated sulphuric acid (96% H_2_SO_4_) was added to the mixture and vortexed. The mixture was then allowed to stand for 10 min and cooled down in a room temperature water bath for 10 min. The mixture was vortexed again and the absorbance of the mixture was measured using microplate reader at 490 nm (Nielsen, 2010). The carbohydrate content in the sample was determined by using a standard graph plotted with glucose. The percentage change in the tests relative to the control was calculated using Eq. (4).

(4) $${\rm{\%}\;change \;in \;carbohydrate\; content = (C_{0} - C_{1}) \times 100\%/C_{0}}$$

where

C_0_ = Carbohydrates in untreated cells

C_1_ = Carbohydrates in treated cells

### Effects of Ag NPs to lipids

The lipid content in the samples were extracted using a modification of Bligh and Dyer method. A volume of 40 mL of cyanobacterial suspension was centrifuged for 10 min at 5,000 rpm and 7.6 mL of chloroform/methanol/water (1/2/0.8, v/v/v) was added to re-suspend the pellet. The mixture was sonicated for 1 min at 200 W and 45 kHz (Tru-Sweep Ultrasonic Cleaner, Crest Ultrasonics, Malaysia) and vortexed for 30 s. Then, a 2 mL of chloroform and water was added to the mixture to make the final ratio of chloroform/methanol/water to 1/1/0.9 (v/v/v). The mixture was vortexed again for 30 s and centrifuged for 5 min at 5,000 rpm to separate the mixture into three layers. The upper methanol layer was removed and the chloroform layer at the bottom that contained lipids was transferred into a new pre-weighted tube. The extraction process was repeated twice with the remaining middle layer. The three chloroform layers were combined in the same tube and evaporated at 80 °C for 24 h in a drying oven (Rizwan, Mujtaba & Lee, 2017). The weight of the lipids was measured and compared to the control (Eq. (5)).

(5) $${\rm{\%\;change \;in\; the \;lipid\; content = (L_{0} - L_{1}) \times 100\%/L_{0}}}$$

where

L_0_ = Lipids in untreated cells

L_1_ = Lipids in treated cells

### Effects of Ag NPs to chlorophyll-*a*, carotenoids, and C-phycocyanin

Chlorophyll-*a* and carotenoids were extracted using 90% methanol and measured spectrophotometrically. A volume of 5 mL of cyanobacterial suspension was centrifuged at 5,000 rpm for 10 min and the supernatant was discarded. A volume of 5 mL of 90% methanol was added to the pellet and vortexed. The mixture was then incubated in a 60 °C water bath for 10 min. The mixture was centrifuged at 5,000 rpm for 5 min to settle down the solid particles. The absorbance of the supernatant was measured using microplate reader at 470, 652, and 665 nm (Kondzior & Butarewicz, 2018). The chlorophyll-*a* and carotenoids content were calculated using the Eqs. (6)–(8). The percentage of change in chlorophyll-*a* and carotenoids in the treatments were calculated by comparing with the untreated cells.

(6) $${\rm Chlorophyll}{-a} \ (\rm C{\it a}) = 16.82A_{665} - 9.28A_{652} (mg/mL)$$

(7) $${\rm Chlorophyll}{-b} {\;(\rm C{\it b}) = 36.92A_{652} - 16.54A_{665} (mg/mL)}$$

(8) $${\rm Carotenoids\; (Ct) = (1,\!000A_{470} - 1.91C{\it a} - 95.15C{\it b})/225 \;(mg/mL)}$$

C-phycocyanin in the samples was extracted using the ultrasonic treatment method. A volume of 5 mL of cyanobacterial sample was centrifuged at 5,000 rpm for 10 min and the collected pellet was washed with 5 mL of distilled water (Akbarnezhad et al., 2016). A volume of 2 mL of 0.05 M phosphate buffer (pH 6.7) and three pieces of glass pearl were added to the washed pellet. The mixture was then vortexed and sonicated in ultrasonic bath for 1 h (Djearamane et al., 2018). After that, the mixture was centrifuged at 5,000 rpm for 5 min and the absorbance of the supernatant was measured at 615 and 652 nm using microplate reader. The C-phycocyanin content in the samples was calculated using Eq. (9) (Bennett & Bogorad, 1973). The percentage of change in phycocyanin was calculated relating to the control sample.

(9) $${\rm C-Phycocyanin\; (C-PC) = (A_{615} - 0.474A_{652})/5.34\;(mg/mL)}$$

### Effects of Ag NPs to total phenolic compounds

The extraction of phenolic compounds from the cells was done using distilled water. A volume of 5 mL of cyanobacterial sample was centrifuged at 5,000 rpm for 10 min and the supernatant was discarded. A volume of 5 mL of distilled water was added to the pellet and incubated in 80 °C water bath for 10 min. The mixture was then cooled down to room temperature and centrifuged at 5,000 rpm for 5 min to settle down solid particle. The total phenolic content in the supernatant was estimated by the Folin-Ciocalteu method (Machu et al., 2015). A volume of 1 mL of supernatant was transferred into a new tube and 1 mL of Folin-Ciocalteau reagent (FCR) and 5 mL of distilled water were added. The mixture was vortexed and allowed to stand for 5 min in dark at room temperature. A volume of 1 mL of 20% Na_2_CO_3_ was added and the solution was then made up to 10 mL with distilled water. The solution was vortexed and again incubated in dark for 1 h at room temperature. The absorbance of the solution was measured using microplate reader at 765 nm (Casazza et al., 2015). Gallic acid was used to plot a standard curve to estimate the total phenolic content in the cyanobacterial samples. The percentage of change in the tests relative to the control was calculated using Eq. (10).

(10) $${\rm\% \;change\; in \;total\; phenolic\; compounds = (P_{0} - P_{1}) \times 100\%/P_{0}}$$

where

P_0_ = Phenolic compounds in untreated cells

P_1_ = Phenolic compounds in treated cells

### Statistical analysis

Statistical analysis was performed to analyze the variance induced by Ag NPs on the algal cells. All the tests in this study were conducted in triplicates (*n* = 3) and the data are presented as mean ± standard deviation. Shapiro-Wilk test was used to test the normal distribution of the data. All data were processed using one-way analysis of variance (ANOVA) followed by Tukey’s *post-hoc* test for multiple comparisons with significance value of *p* < 0.05.

## Results and discussion

### Characterization of Ag NPs

**Figure 1:**
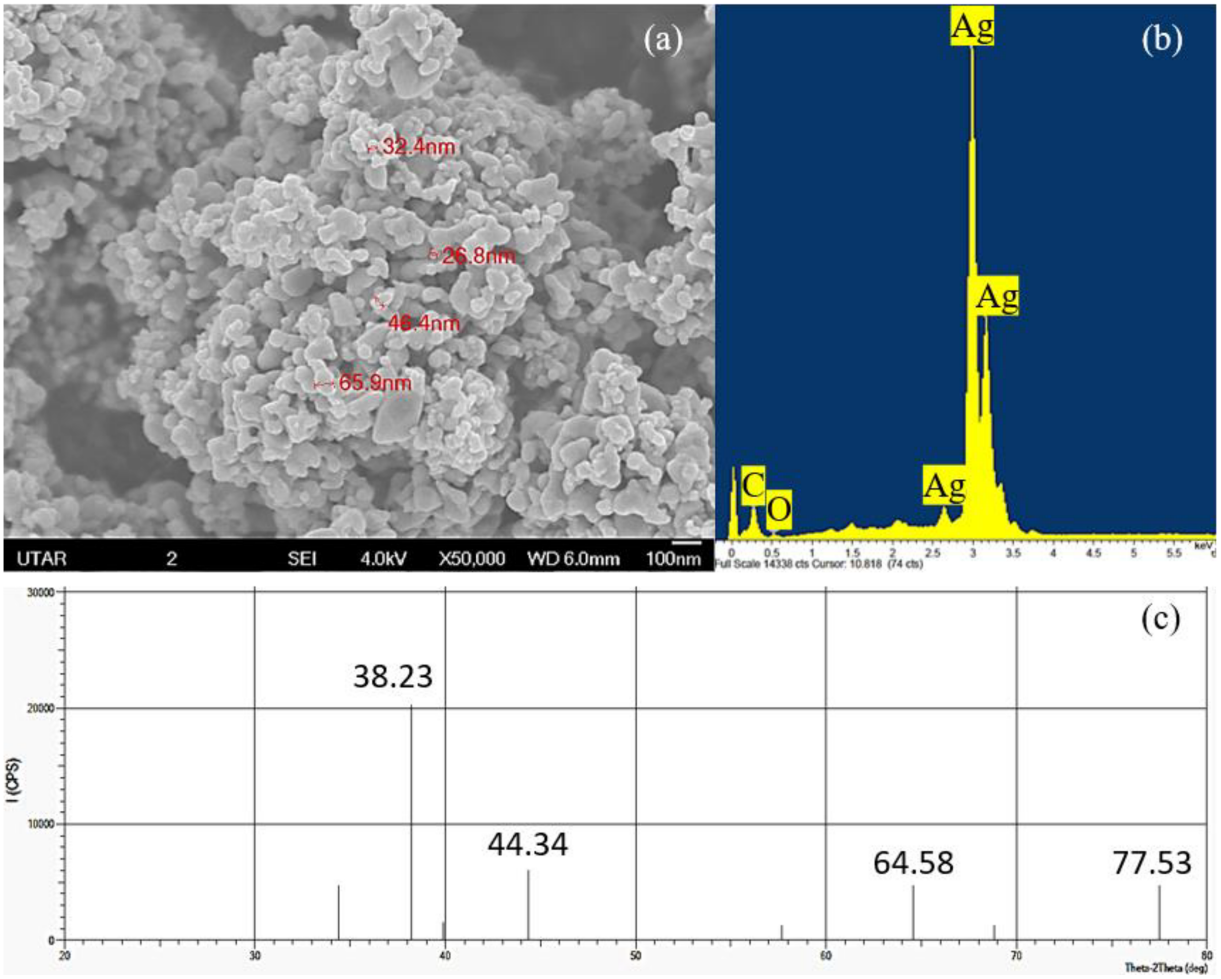
Characterization of Ag NPs. (A) SEM with 50,000× magnification showing the size of the Ag NPs, (B) EDX spectrum and (C) XRD pattern that confirmed the presence of Ag.

The elemental component and crystalline nature of Ag NPs was confirmed using EDX and XRD while the shape and size of Ag NPs were observed through SEM. Based on the SEM observation, cubic shaped NPs were observed in the agglomerated state and the average size of the particles was measured to be 43.38 nm with a range of 26.8 to 65.9 nm (Fig. 1A). The EDX spectrum showed the presence of silver element in the Ag NPs powder studied (Fig. 1B) and the XRD pattern confirmed the cubic structure and the light gray metallic colour of Ag NPs (Fig. 1C).

### Determination of cellular interaction of Ag NPs on the cells

**Figure 2:**
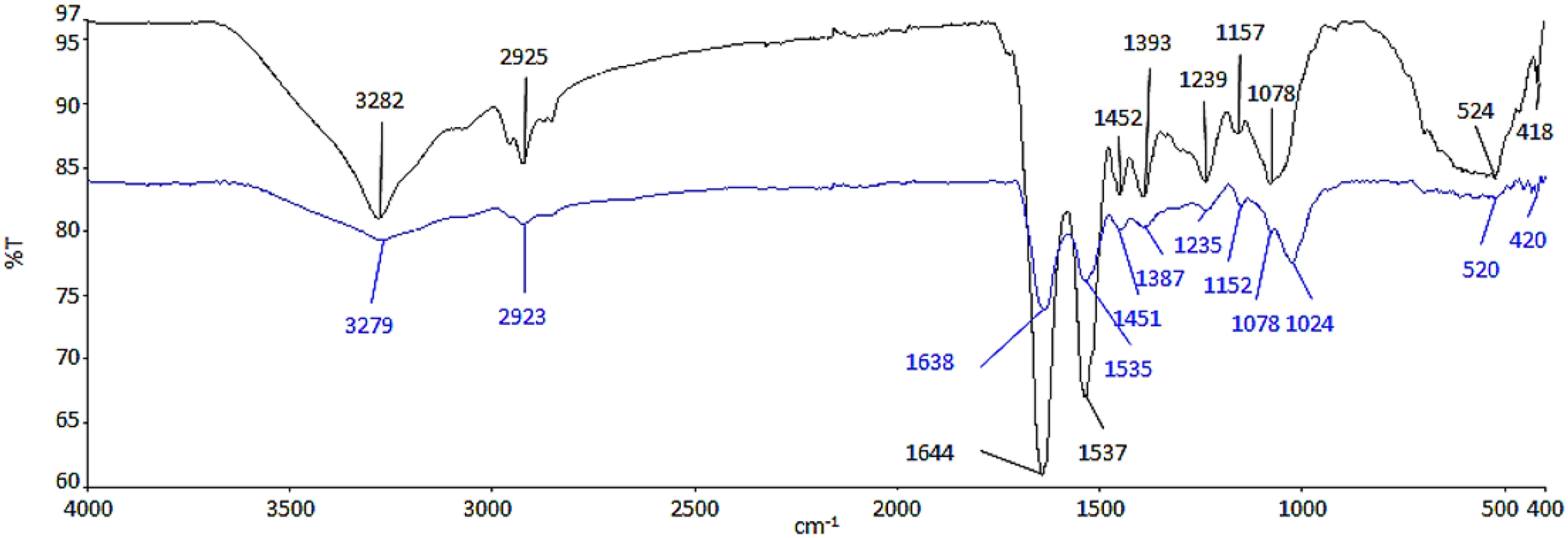
ATR-FTIR spectrum of control (black line) and 100 μg/mL Ag NPs treated *A*. *platensis* at 96 h (blue line).

ATR-FTIR analysis was conducted to determine the functional groups from the biomolecules of cell wall, which had possibly interacted with Ag NPs (Fig. 2). The functional groups and biomolecules which were possibly involved in the surface binding and accumulation of NPs on the cell surface are listed in Table 1.

**Table 1:** Possible involvement of functional groups in ATR-FTIR.

Absorption (cm^−1^)	Functional groups	Component
3,282 → 3,279	−OH, −NH	Hydroxyl, amine (protein)
2,925 → 2,923	−CH_2_	Methyl (lipid fraction)
1,644 → 1,638	C=O	Amide I (protein)
1,537 → 1,535	N–H, C–N	Amide II (protein)
1,393 → 1,387	C=O	Carbonyl (aldehyde, ketone, carboxylate)
1,239 → 1,235	P=O	Phosphodiesters (nucleic acid, phospholipids)
1,157 → 1,152	C–O, C–C	Carbohydrate
1,024	C–C, C–O, C–OH	Carboxyl, hydroxyl
524 → 520	−PO, −CH	Phosphate

The region between 3,282 and 3,279 cm^−1^ is relative to the symmetric –OH and –NH stretching of the hydroxyl and amide functional groups from water and protein (Ansari et al., 2019; Dotto, Cadaval & Pinto, 2012; Zinicovscaia et al., 2020). The presence of asymmetric –CH_2_ stretching vibration of methyl group was found between 2,925 and 2,923 cm^−1^, which belongs to the long methylenic chains of lipidic fractions (Ansari et al., 2019; Ferreira et al., 2011). The peaks at 1,644 and 1,639 cm^−1^ were the symmetric C=O stretching of protein amide I, while, the symmetric deformation of N–H bend and C–N stretching of protein amide II can be found between 1,537 and 1,535 cm^−1^ (Ansari et al., 2019; Bataller & Capareda, 2018). The region between 1,393 and 1,387 cm^−1^ could be attributed to C=O stretching of aldehydes, ketones, and carboxylate (Çelekli, Gün & Bozkurt, 2021; Çelekli, Yavuzatmaca & Bozkurt, 2010). Peaks at 1,239 and 1,235 cm^−1^ were linked to asymmetrical P=O stretching of phosphodiester of nucleic acids and phospholipids (Ansari et al., 2019; Fang et al., 2011). Also, the region between 1,157 and 1,152 cm^−1^ was assigned as the carbohydrate characteristic bands with C–O and C–C stretching (Bataller & Capareda, 2018; Ferreira et al., 2011). In contrast to the control cell, the peak at 1,078 cm^−1^ which indicates the CO stretching of alcoholic group (Sheng et al., 2004) was shifted and formed a new band at 1,024 cm^−1^ in the Ag NPs treated cell representing C–O, C–C, and C–OH stretching of the carboxyl and hydroxyl groups (Çelekli, Yavuzatmaca & Bozkurt, 2010; Zinicovscaia et al., 2020). The peaks between 524 and 520 cm^−1^ were the –PO and aromatic –CH stretching of phosphate (Zinicovscaia et al., 2020).

Based on the results in Fig. 2, there was a decrease in the relative intensity of the OH bands in the Ag NPs treated cells and also the NH bands, suggesting the interaction of ions with NH groups through the electron lone pairs of the nitrogen atom (Ferreira et al., 2011). The decrease in the relative intensity of peaks between 1,638 and 1,387 cm^−1^ demonstrated the involvement of amide and carboxyl groups in the adsorption of NPs where it may be due to the interaction of the cation with the amide group, characterized by electron lone pairs over oxygen and nitrogen atoms. The regions corresponding to the carbohydrate and phosphate functional groups did not seem to be significantly affected by the adsorption of NPs (Ferreira et al., 2011).

**Figure 3:**
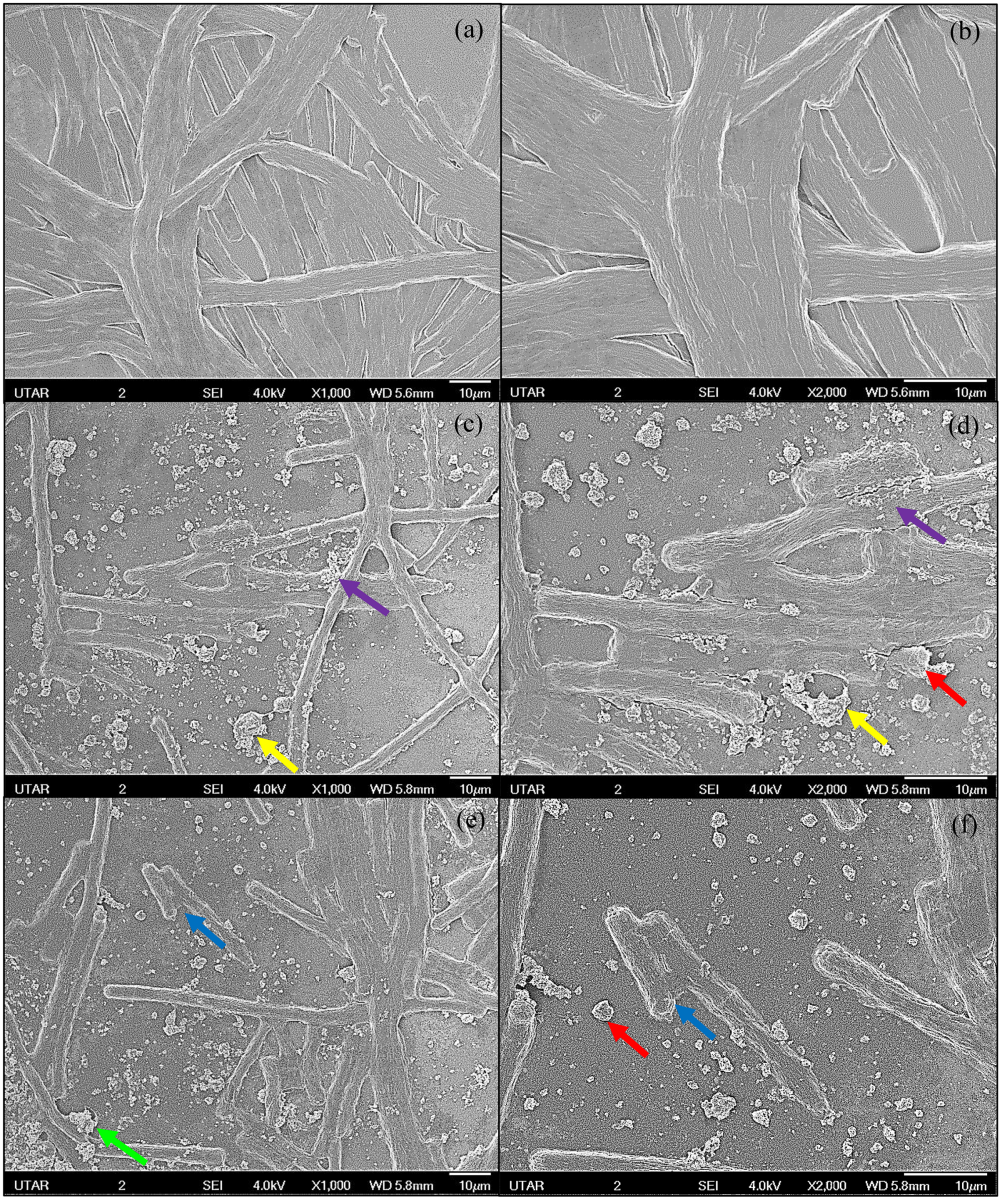
SEM images of control and 100 μg/mL Ag NPs treated *A. platensis* at 96 h. (A) control at 1,000× magnification; (B) control at 2,000× magnification; (C, E) 100 μg/mL Ag NPs treated *A. platensis* at 1,000× magnification; (D, F) 100 μg/mL Ag NPs treated *A. platensis* at 2,000× magnification. The agglomeration of Ag NPs (green arrow), short fragments of *A. platensis* cells (blue arrow), the attachment of Ag NPs on *A. platensis* (purple arrow), aggregates of distorted cells with Ag NPs (yellow arrow), and the breakage of *A. platensis* cells (red arrow) can be clearly observed.

**Figure 4:**
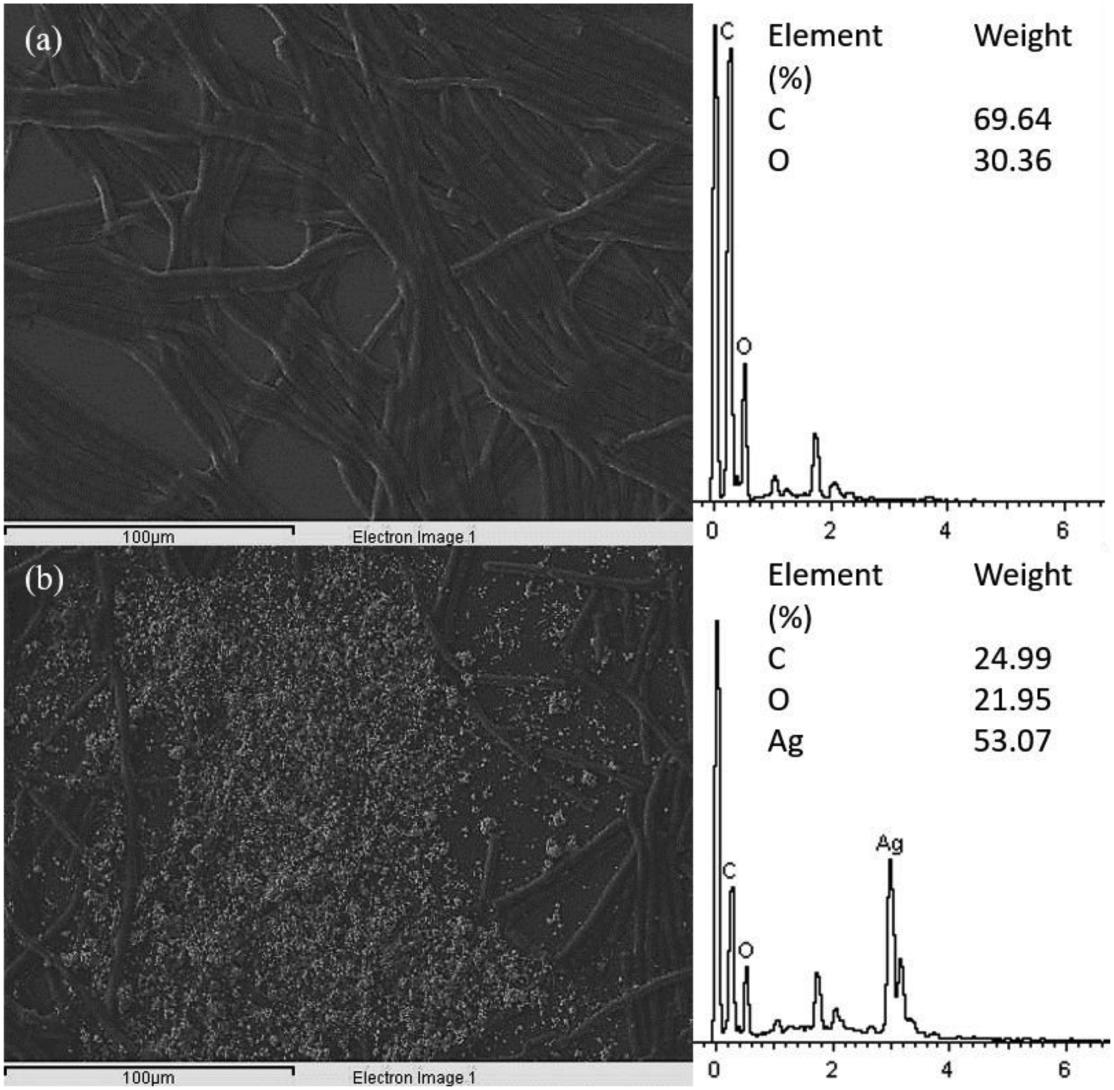
EDX image and spectrum of control (A) and 100 μg/mL Ag NPs treated *A. platensis* at 96 h (B).

The SEM images of control and 100 μg/mL Ag NPs treated *A. platensis* cells are shown in Fig. 3. The control cells showed the smooth cylindrical cells with intact cell membrane, while the cells treated with Ag NPs showed the attachment of NPs on cell surface, fragmentation and distortion of *Arthrospira* cells. The EDX spectrum of Ag NPs treated cells (Fig. 4) showed the presence of Ag, which was not present in the control cells. The results confirmed the accumulation of Ag NPs on *A. platensis*. Similar findings were reported on *A. platensis* treated with zinc oxide nanoparticles (ZnO NPs) (Djearamane et al., 2018) and *Skeletonema costatum* treated with Ag NPs (Huang, Cheng & Yi, 2016). The physical barrier formed during the surface attachment of NPs on the cells was reported to inhibit the growth of photosynthetic microbes. The adsorption and accumulation of multiple layers of NPs on the cell surface might affect the transfer of nutrients and induce physical stress to the photosynthetic microbes (Metzler et al., 2011). The pore diameter in the cell wall of microalgae was found to be ranging from 5 to 20 nm (Navarro et al., 2008), which is much smaller than the Ag NPs size used in this study and hence it is assumed that the Ag NPs were mainly adsorbed on the surface of *A. platensis*. The agglomeration of Ag NPs on the cell surface was found to play a vital role in the toxicity of Ag NPs to the photosynthetic microbes grew in salt water (Sendra et al., 2017). The adsorption and aggregation of NPs on the cell surface can cause mechanical damage and affect the cellular metabolism (Hazeem et al., 2016) and thus can cause the growth inhibition and the corresponding reduction in biomass and photosynthetic pigments (Metzler et al., 2011).

### Effects of Ag NPs to biomass

**Figure 5:**
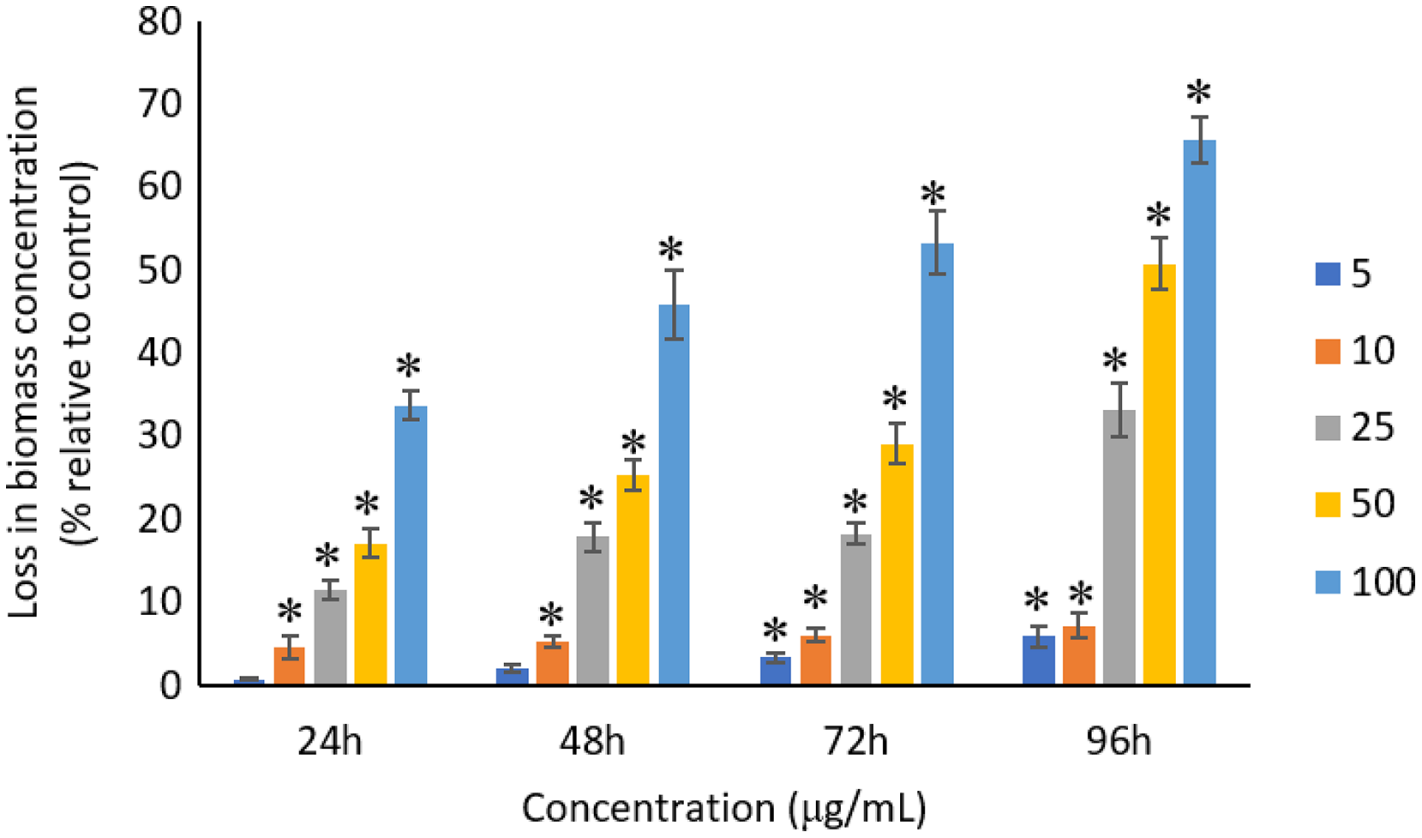
Percentage of loss in biomass concentration of *A. platensis* relative to control from 24 to 96 h upon treatment of Ag NPs. The values plotted are in mean ± standard deviation. An asterisk (*) indicates the significance difference at *p* < 0.05 between the control and Ag NPs treated cyanobacteria suspension for each time interval.

The exposure of Ag NPs caused significant (*p* < 0.05) loss in the biomass of *A. platensis* for concentrations ≥10 μg/mL at 24 and 48 h. While, significant (*p* < 0.05) loss in biomass was observed for all tested concentrations from 5 to 100 μg/mL at 72 and 96 h. The maximum loss in biomass of *A. platensis* happened in 96 h with the reported values of 5.86 ± 1.21%, 7.21 ± 1.51%, 33.13 ± 3.30%, 50.79 ± 3.22%, and 65.71 ± 2.79% for 5, 10, 25, 50, and 100 μg/mL of Ag NPs, respectively (Fig. 5).

A concentration- and time-dependent loss was observed in the biomass concentration of *A. platensis* due to the exposure of Ag NPs. Studies conducted by Lone et al. (2013) and Comotto et al. (2014) confirmed the loss of biomass of *A. platensis* using different NPs. It was reported that 10 μg/mL of 50 nm ZnO NPs caused 41% loss in biomass concentration of *A. platensis* when exposed for 10 days (Lone et al., 2013), and 74% loss in biomass concentration when treated with 100 μg/mL of 14 nm TiO_2_ NPs for 15 days (Comotto et al., 2014). Previous studies have presented the growth inhibitory effect of Ag NPs on different photosynthetic microbes. For instance, 200 μg/mL of Ag NPs can cause a complete inhibition in *Scenedesmus* sp. and almost 100% inhibition in *Thalassiosira* sp. at 72 h with the EC_50_ values of 89.92 and 107.21 μg/L for *Scenedesmus* sp. and *Thalassiosira* sp., respectively (Pham, 2019). Smaller size Ag NPs was found to cause higher growth inhibition on *Chlamydomonas reinhardtii* and as *C. reinhardtii* treated with 10 μg/L of Ag NPs with size of 4.5 and 16.7 nm showed more than 50% growth inhibition at 72 h with the EC_50_ value of <10 μg/L for Ag NPs, whereas the EC_50_ value of >300 μg/L was reported for Ag NPs with 46.7 nm (Sendra et al., 2017). Djearamane et al. (2019) evidenced a significant loss in cell viability and the corresponding biomass of *C. vulgaris* when treated with ZnO NPs.

The growth rate of photosynthetic microbes exposed to Ag NPs might be affected by the shape, size, concentration, surface charge and also surface coatings (Cepoi et al., 2020). Many researchers suggested that the growth inhibition of cells was due to the generation of reactive oxygen species (ROS) (Suman, Radhika Rajasree & Kirubagaran, 2015; Xia et al., 2015; Djearamane et al., 2020) or the mechanical damage caused by NPs on the cells (Castro-Bugallo et al., 2014). Other factors such as the light shading effect (Sadiq et al., 2011), release of metal ions (Aravantinou et al., 2015; Lee & An, 2013; Suman, Radhika Rajasree & Kirubagaran, 2015), interaction with the culture medium (Manier et al., 2013), and the synergistic effects of these different factors (Manzo et al., 2013) were also reported to affect the growth of the cells. The destruction of cell membrane upon exposure to NPs is one of the factors that cause the growth inhibition, which leads to the uncontrolled release and intake of electrolytes and subsequently affect the photosynthesis apparatus and also the synthesis of macronutrients (Anusha, Chingangbam & Sibi, 2017). The interaction of metal ions with the functional groups present on the surface of the cells would affect the growth rate as well (Balaji, Kalaivani & Rajasekaran, 2014).

### Effects of Ag NPs to proteins, carbohydrates and lipids

**Figure 6:**
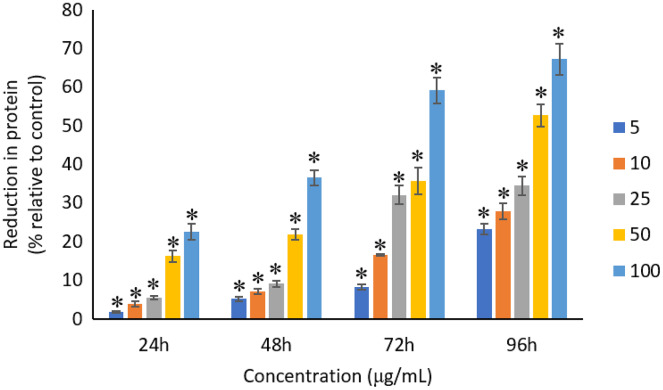
Percentage of loss in proteins of *A. platensis* relative to control from 24 to 96 h upon treatment of Ag NPs. The values plotted are in mean ± standard deviation. An asterisk (*) indicates the significance difference at *p* < 0.05 between the control and Ag NPs treated cyanobacteria suspension for each time interval.

**Figure 7:**
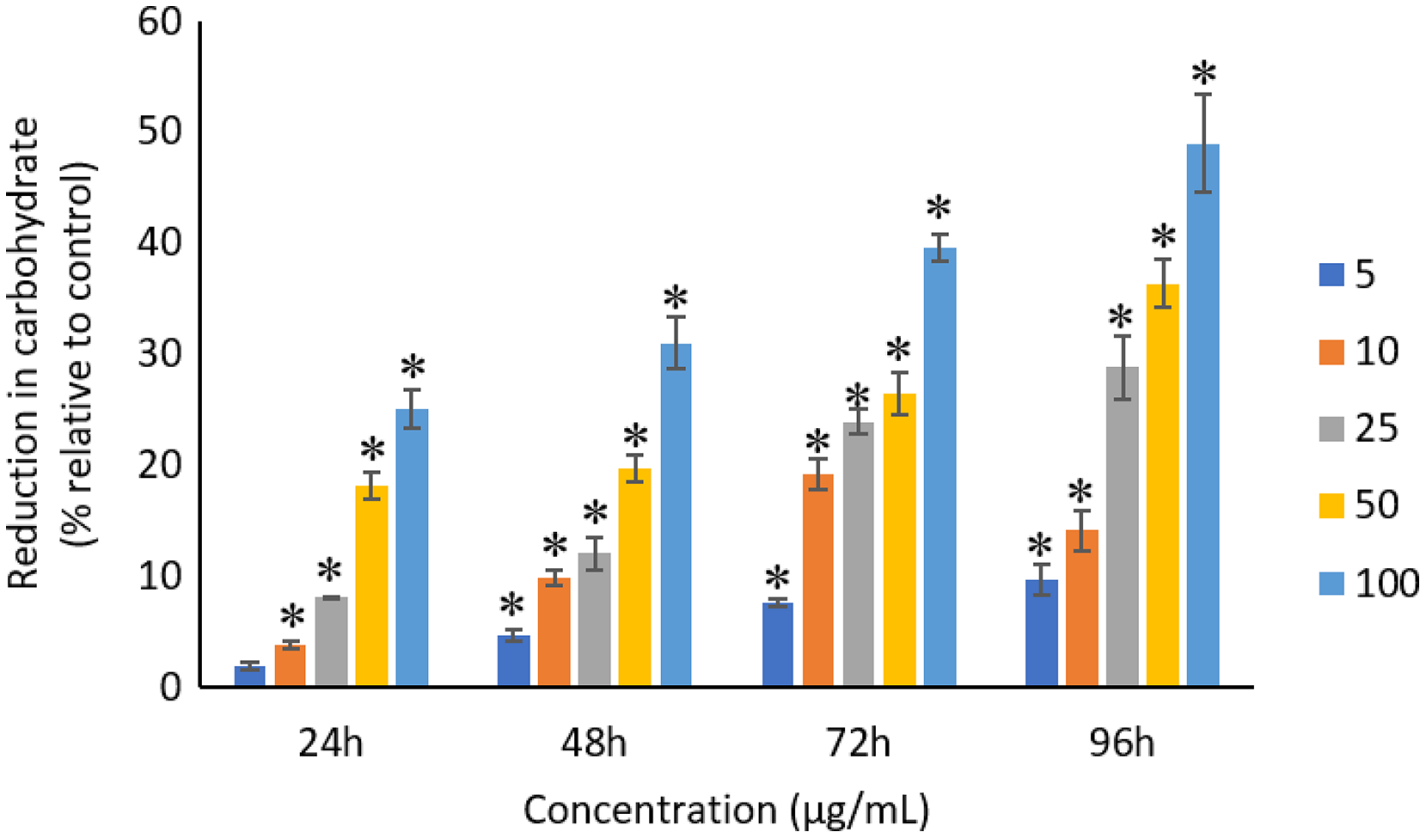
Percentage of loss in carbohydrates of *A. platensis* relative to control from 24 to 96 h upon treatment of Ag NPs. The values plotted are in mean ± standard deviation. An asterisk (*) indicates the significance difference at *p* < 0.05 between the control and Ag NPs treated cyanobacteria suspension for each time interval.

**Figure 8:**
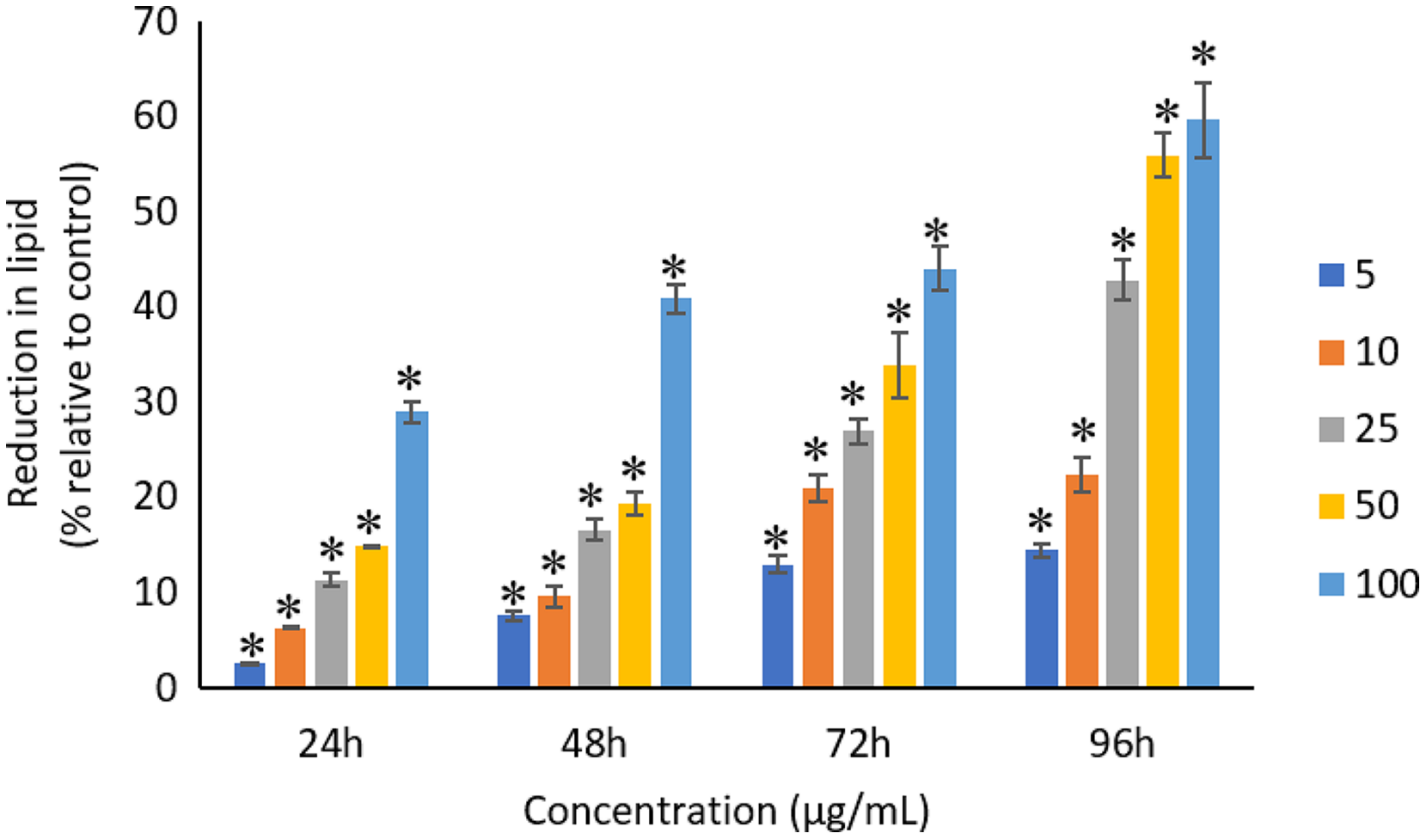
Percentage of loss in lipids of *A. platensis* relative to control from 24 to 96 h upon treatment of Ag NPs. The values plotted are in mean ± standard deviation. An asterisk (*) indicates the significance difference at *p* < 0.05 between the control and Ag NPs treated cyanobacteria suspension for each time interval.

All the tested concentrations of Ag NPs caused significant (*p* < 0.05) loss in the protein content of *A. platensis* from 24 to 96 h (Fig. 6). Maximum reduction in protein content was observed at 96 h with the resultant values of 23.27 ±1.43%, 27.83 ± 2.10%, 34.45 ± 2.40%, 52.65 ± 2.98%, and 67.21 ± 3.98% for 5, 10, 25, 50, and 100 μg/mL of Ag NPs, respectively. The exposure of Ag NPs caused significant (*p* < 0.05) loss in carbohydrates of *A. platensis* for the concentrations ≥10 μg/mL at 24 h. While, significant (*p* < 0.05) was observed in all the tested concentrations of Ag NPs from 48 to 96 h (Fig. 7). Maximum reduction in carbohydrate content was observed at 96 h with the resultant values of 9.68 ± 1.39%, 14.07 ± 1.77%, 28.78 ± 2.82%, 36.35 ± 2.10%, and 48.99 ± 4.39% for 5, 10, 25, 50, and 100 μg/mL of Ag NPs, respectively, while the significant (*p* < 0.05) loss in lipids was observed from 24 to 96 h for all concentrations tested (Fig. 8). Maximum reduction in lipid content was observed at 96 h with the resultant values of 14.39 ± 0.78%, 22.34 ± 1.88%, 42.76 ± 2.09%, 55.92 ± 2.31%, and 59.62 ± 3.96% for 5, 10, 25, 50, and 100 μg/mL of Ag NPs, respectively.

A dose and time-dependent decrease in protein content observed from this study may be due to the stress caused by Ag NPs present in the culture medium (Holan & Volesky, 1994). Similar results were shown when *A. platensis* was treated with different heavy metals and NPs. *A. platensis* treated with 10 μg/mL ZnO NPs was reported to have a 79% reduction in protein content when exposed for 10 days (Lone et al., 2013), while 32%, 64% and 69% reduction was observed when treated with 100 μg/mL of Se metal at 24, 48 and 72 h (Zinicovscaia et al., 2017). Balaji, Kalaivani & Rajasekaran (2014) reported that the exposure of 42 mg/L of Ni and 48 mg/L of Zn to *A. platensis* for 18 days led to the highest reduction in protein content.

Similar to proteins, a dose and time-dependent reduction in the carbohydrates of *A. platensis* was observed in this study. A study by Zinicovscaia et al. (2017) reported 76% reduction in the carbohydrate content of *A. platensis* when treated with 100 μg/mL of Se at 72 h. However, another study on *S. costatum* using 0.05 to 50 ug/mL of Ag NPs evidence only a limited impact on the carbohydrate content when exposed for 24 h (Huang, Cheng & Yi, 2016).

The study of Zinicovscaia et al. (2017) reported that 100 μg/mL of Se metal caused 23%, 50% and 80% reduction in lipid content of *A. platensis* when exposed to 24, 48 and 72 h, respectively. *A. platensis* treated with 100, 250 and 500 μg/mL of TiO_2_ NPs stated to have 44%, 53% and 66% reduction in lipid yield at Day 5 (Casazza et al., 2015). On the other hand, Pham (2019) reported that Ag NPs at the concentrations of 5 to 200 μg/mL would raise the lipid yield from 11% to 17% at 72 h for *Thalassiosira* sp. The lipid content in *Scenedesmus* sp. also showed increment of 8.1% and 7.6% with the presence of Ag NPs at the concentrations of 5 and 20 μg/mL, and then decreased at higher concentrations of 100 and 200 μg/mL.

A study investigated the gene expression of *C. reinhardtii* when exposed to silver and reported that most of the protein were significantly regulated, however, only observed at the transcriptome but not at the proteome level, which may be the reason for the reduced synthesis of protein in microalgae (Pillai et al., 2014). The treatment of TiO_2_ NPs was also reported to disrupt the material and energy metabolism in *Chlorella pyrenoidosa* at the molecular level, where the gene expression of lipid synthesis, carbohydrate synthesis and cell division were down regulated (Middepogu et al., 2018), indicating the inhibition of biosynthesis of lipid and carbohydrate and also the cell division of *C. pyrenoidosa* at the gene expression level. The generation of ROS and the release of metal from the treatment of NPs induce the oxidation of the functional groups and structural components of cyanobacteria and subsequent degradation of the proteins, lipids and carbohydrates into smaller molecules up to monomers, small inorganic molecules and water, and, consequently, biomass degradation (Khalifeh, Salari & Zamani, 2022; Zinicovscaia et al., 2017). ROS can also lead to the subsequent lipid peroxidation which will interrupt the cell metabolism (Wang et al., 2019). Cyanobacteria have the tendency to adjust cellular metabolism through reduction in the biosynthesis of metabolites under stress condition when exposed to NPs (Pham, 2019; Khalifeh, Salari & Zamani, 2022).

The reduction in all these biological macromolecules of photosynthetic microbes might be due to the oxidative stress induced by the heavy metals or NPs that are present in the culture medium. A study reported that the cells that are growing under stress conditions were observed to have a lower protein synthesis capacity (Zeng & Vonshak, 1998). The high concentrations of NPs used during the exposure to the cells caused the damage in the cell membrane that led to the reduction in the synthesis of carbohydrates, which subsequently caused the uncontrolled release and intake of electrolytes (Anusha, Chingangbam & Sibi, 2017). The decrease in carbohydrates may also be affected by the production of chlorophyll as carbohydrates are the main photosynthesis products that are stored in the chloroplasts (Huang, Cheng & Yi, 2016). The change in the lipid content was due to the response to stress based on the condition and alteration in the physiological state of the cells as the phospholipids in the cell membrane play an important role in metabolism (Zinicovscaia et al., 2017). In short, the binding of NPs on the cell membrane leads to the damage in the cell membrane which poses a vital role in reducing the production of proteins, carbohydrates and lipids in the photosynthetic microbes.

### Effects of Ag NPs to chlorophyll-*a*, carotenoids, and C-phycocyanin

**Figure 9:**
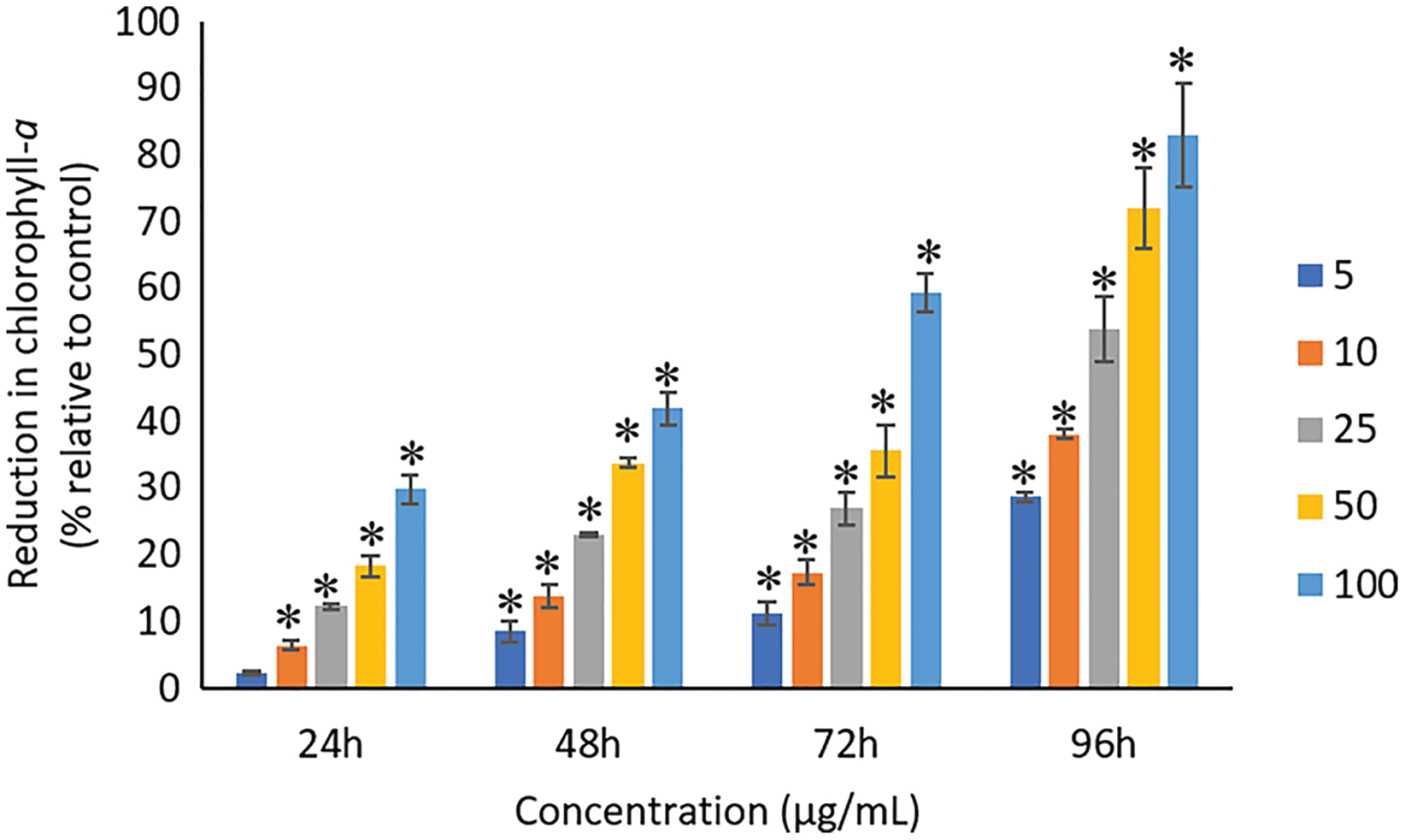
Percentage of loss in chlorophyll-*a* of *A. platensis* relative to control from 24 to 96 h upon treatment of Ag NPs. The values plotted are in mean ± standard deviation. An asterisk (*) indicates the significance difference at *p* < 0.05 between the control and Ag NPs treated cyanobacteria suspension for each time interval.

**Figure 10:**
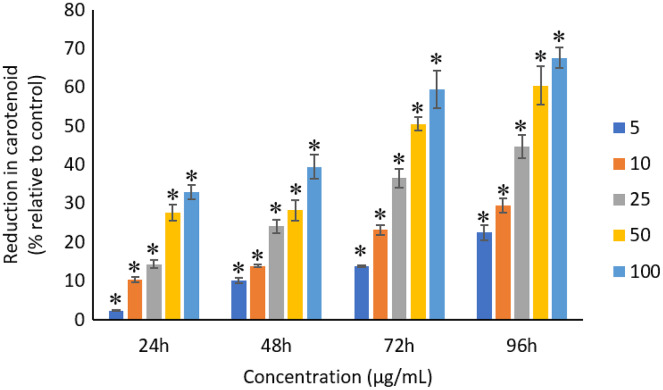
Percentage of loss in carotenoids of *A. platensis* relative to control from 24 to 96 h upon treatment of Ag NPs. The values plotted are in mean ± standard deviation. An asterisk (*) indicates the significance difference at *p* < 0.05 between the control and Ag NPs treated cyanobacteria suspension for each time interval.

**Figure 11:**
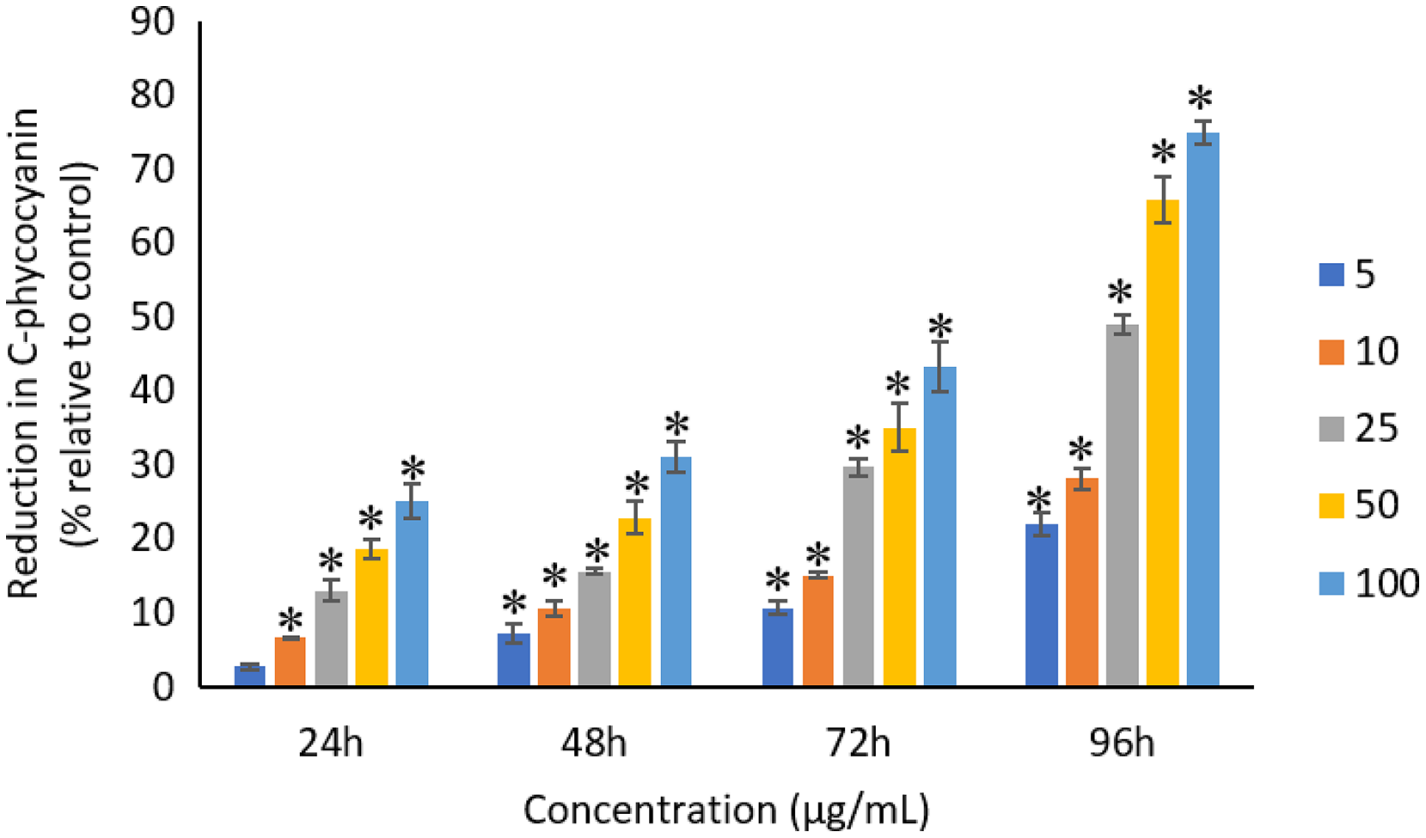
Percentage of loss in C-phycocyanin of *A. platensis* relative to control from 24 to 96 h upon treatment of Ag NPs. The values plotted are in mean ± standard deviation. An asterisk (*) indicates the significance difference at *p* < 0.05 between the control and Ag NPs treated cyanobacteria suspension for each time interval.

The exposure of Ag NPs caused significant (*p* < 0.05) loss in chlorophyll-*a* of *A. platensis* for concentrations ≥10 μg/mL at 24 h. While, significant (*p* < 0.05) loss was observed in all the tested concentrations of Ag NPs from 48 to 96 h (Fig. 9). Maximum reduction in chlorophyll-*a* content was observed at 96 h with the resultant values of 28.63 ± 0.83%, 38.10 ± 0.70%, 53.81 ± 4.83%, 71.97 ± 5.94%, and 82.99 ± 7.81% for 5, 10, 25, 50, and 100 μg/mL of Ag NPs, respectively. All the tested concentrations of Ag NPs caused significant (*p* < 0.05) loss in the carotenoids of *A. platensis* from 24 to 96 h (Fig. 10). Maximum reduction in carotenoid content was observed at 96 h with the resultant values of 22.44 ± 2.02%, 29.53 ± 1.84%, 44.67 ± 2.94%, 60.46 ± 4.95%, and 67.55 ± 2.63% for 5, 10, 25, 50, and 100 μg/mL of Ag NPs, respectively. The exposure of Ag NPs caused significant (*p* < 0.05) loss in C-phycocyanin of *A. platensis* for concentrations ≥10 μg/mL at 24 h. While, significant (*p* < 0.05) loss was reported in all the tested concentrations of Ag NPs from 48 to 96 h (Fig. 11). Maximum reduction in C-phycocyanin content was observed at 96 h with the resultant values of 21.97 ± 1.57%, 28.18 ± 1.44%, 48.91 ± 1.28%, 65.85 ± 3.01%, and 75.03 ± 1.55% for 5, 10, 25, 50, and 100 μg/mL of Ag NPs, respectively.

A concentration and dose-dependent reduction in the chlorophyll-*a* content of *A. platensis* is related to the stress caused by Ag NPs (Holan & Volesky, 1994). Similar to the present study, a study by Djearamane et al. (2018) reported 63%, 75%, 86%, 88% and 93% reduction in chlorophyll-*a* of *A. platensis* when exposed to 10, 50, 100, 150 and 200 μg/mL of ZnO NPs, respectively at 96 h. A study by Pham (2019) showed 21.5% and 14.5% reduction in chlorophyll-*a* of *Scenedesmus* sp. and *Thalassiosira* sp. respectively, when treated with 200 μg/mL Ag NPs at 72 h. Another study reported that the treatment of 0.05 μg/mL Ag NPs significantly increased the production of chlorophyll-*a* in *S. costatum* by 4.7% at 24 h, and decreased by 2.4% when the concentration of Ag NPs increased to 5 μg/mL. The maximum reduction in chlorophyll-*a* content of *S. costatum* was reported to be 35% when exposed to 500 μg/mL of Ag NPs for 24 h (Huang, Cheng & Yi, 2016).

Like chlorophyll-*a*, the carotenoids and C-phycocyanin exhibited a concentration and time-dependent reduction when exposed to Ag NPs. Similar to the present results, Djearamane et al. (2018) reported a maximum reduction of 76.2% in carotenoids and 74.1% in C-phycocyanin when treated with 200 μg/mL of ZnO NPs, respectively, at 96 h. Another study stated that 10 μg/mL of ZnO NPs caused 50% carotenoids reduction in *A. platensis* when exposed for 10 days (Lone et al., 2013). While, Zinicovscaia et al. (2017) reported that the C-phycocyanin content of *A. platensis* reduced from 6.9% to 2.4% at 24 h when exposed to Se ions. The lowest C-phycocyanin content was observed at 72 h with 0.66% C-phycocyanin in *Arthrospira* biomass, which contained only about 10% of the original amount.

Chlorophyll-*a* is a useful indicator to determine the efficiency of photosynthesis and growth status of photosynthetic microbes. The inhibition of the biosynthesis of the key protein for photosynthesis might further affect the photosynthesis products (Huang, Cheng & Yi, 2016). The reduction of chlorophyll-*a* may be due to the damage in chloroplast ribosomes (Sendra et al., 2017), increased activity of chlorophyllase, the disruption of membrane system, and also the inactivation of electron transport functions in the photosystem (Holan & Volesky, 1994). The effective quantum yield of PSII was decreased in cyanobacteria when treated with NPs marks the decreased efficiency of the photochemical energy conversion process (Sendra et al., 2017). Carotenoids are the accessory photosynthetic pigments that primarily absorbs light in the blue-green region (Miazek et al., 2015). While, C-phycocyanin is a type of phycobilins that are present in cyanobacteria which helps to absorb the wavelengths of light between 595 and 640 nm to supplement the light-capturing ability of chlorophyll (Frank & Cogdell, 2012). The reduction in the accessory photosynthetic pigments (carotenoids and C-phycocyanin) under stress conditions affects the light-absorbing ability of chlorophyll which subsequently leads to the inhibition of growth. The exposure of NPs to cyanobacteria also leads to the destruction of thylakoids that causes the decreased of photosynthetic pigments which affects the photosynthesis of cyanobacteria, resulting in the inhibition of growth and cell death (Arunakumara & Zhang, 2008). The attachment of Ag NPs on the surface of the cells can cause the physical shading and act as a photosynthesis barrier which in turn can result in the loss of biomass (Huang, Cheng & Yi, 2016) and thus the macronutrients.

### Effects of Ag NPs to total phenolic compounds

**Figure 12:**
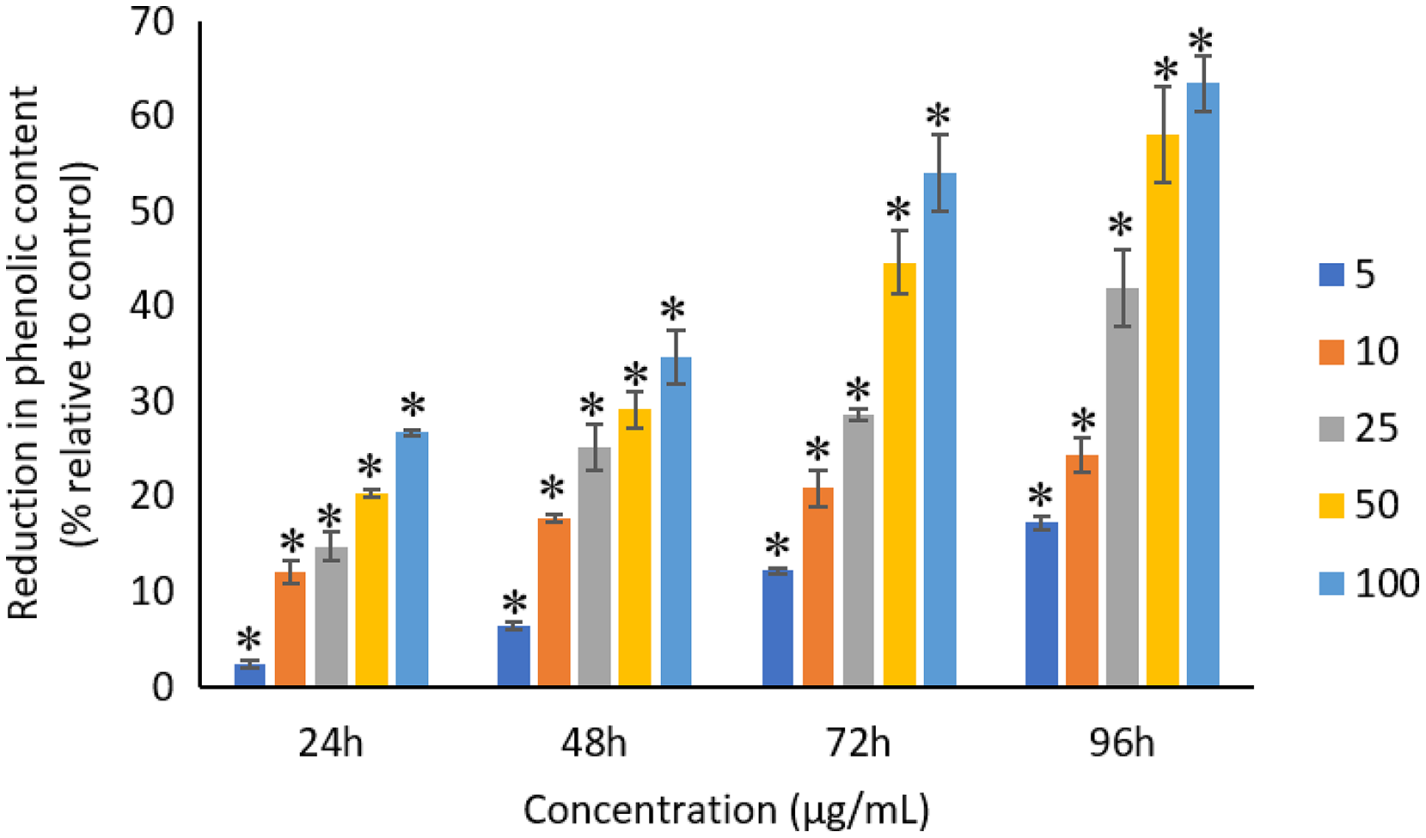
Percentage of loss in total phenolic compounds of *A. platensis* relative to control from 24 to 96 h upon treatment of Ag NPs. The values plotted are in mean ± standard deviation. An asterisk (*) indicates the significance difference at *p* < 0.05 between the control and Ag NPs treated cyanobacteria suspension for each time interval.

All the tested concentrations of Ag NPs caused significant (*p* < 0.05) loss in the total phenolic compounds of *A. platensis* from 24 to 96 h (Fig. 12). Maximum reduction in phenolic content was observed at 96 h with the resultant values of 17.27 ± 0.65%, 24.29 ± 1.82%, 41.96 ± 4.04%, 58.10 ± 4.99%, and 63.43 ± 2.89% for 5, 10, 25, 50, and 100 μg/mL of Ag NPs, respectively.

A typical dose-dependent and time-dependent reduction was observed for the total phenolic contents of *A. platensis* when treated with Ag NPs. Earlier study by Fazelian et al. (2020) reported a significant decrease in phenolic compounds of *Nannochloropsis oculate* when exposed to 25 to 50 mg/L of Ag NPs, although all the concentrations tested in the study showed decrease in the phenolic compound even from 1 mg/L of Ag NPs. In another study, *C. vulagris* cells treated with 2 to 6 mg/L of microwave synthesized silver-reduced graphene oxide nanocomposites (Ag-rGO) were also reported to have significant decrease in the phenolic contents at 24 h as compared to the control (Nazari et al., 2020). Similar reduced production of phenolic content was also found when using hydrothermal synthesized Ag-rGO (Nazari et al., 2018). Casazza et al. (2015) reported that the *A. platensis* exposed to 100, 250 and 500 μg/mL of TiO_2_ NPs caused 34.9%, 27.9% and 24.5% reduction in intracellular phenolic content at Day 5. Another study reported 12%, 17% and 24% reduction in the intracellular phenolic content of *Haematococcus pluvialis, Chlorella vulgaris* and *A. platensis*, respectively when treated with 100 μg/mL of TiO_2_ NPs at Day 15 as compared to the control (Comotto et al., 2014). Zaidi et al. (2015) also reported a decrease in the total phenolic compounds of *Chlorella* sp. when treated with 5 and 10 ppm of Ag NPs.

Photosynthetic microbes under stress conditions are likely to excrete compounds such as phenolic compounds which can detoxify the surrounding environment and act as an antioxidant. Phenolic compounds are the secondary plant metabolites that are synthesized through the shikimic acid pathway and phenyl propanoid metabolism when they are under optimal conditions, however, different environmental stresses to the cells may change the phenolic content quantity (Vogt, 2010). The increased production of phenolic compounds is stimulated by the cells in response to the stress conditions and released to the culture medium with a decrease in the intracellular concentration of phenolic compounds and increase in the extracellular phenolic content (Comotto et al., 2014). The reduction in total phenolic compounds in the photosynthetic microbes under the exposure of heavy metals may be due to the inhibition of photosynthesis which results in the reduced the synthesis of new phenolic compounds (Connan & Stengel, 2011). The decreased production of the total phenolic compounds can lead to a decreased antioxidant activity which can cause reduction in the biomass (Bello-Bello et al., 2017; Fazelian et al., 2020).

## Conclusion

The exposure of Ag NPs to *A. platensis* resulted in a concentration and time-dependent reduction in biomass, proteins, carbohydrates, lipids, chlorophyll-*a*, carotenoids, C-phycocyanin, and phenolic compounds. The hydroxyl, amine, amides, carbonyl, phosphodiesters and phosphates from the carbohydrates, proteins and phospholipids of the cell wall were found to be responsible for the attachment and accumulation of Ag NPs on the cell surface of *A. platensis*. The interaction between Ag NPs and the functional groups on the cell wall can form a physical barrier and block the photosynthesis activity, the transfer of nutrients, and induced stress to the cells, which led to the growth inhibition and the corresponding reduction in biomass, macromolecules, pigments, and phenolic compounds. The findings of this study may be useful to design methods to detect the contamination of Ag NPs with *A. platensis* and thus will help to produce and provide quality *Arthrospira* nutritional supplements with uncompromised nutritional values to the consumers. Otherwise, the intake of Ag NPs contaminated *A. platensis* supplements might not provide the expected nutritional benefits to the consumers and also could cause health hazards due to the Ag NPs contamination.

## Supplemental Information

10.7717/peerj.13972/supp-1 Supplemental Information 1 Raw data for SPSS.

## Additional Information and Declarations

### Data Availability

The following information was supplied regarding data availability: The raw measurements are available in the Supplemental File.
